# Prognostic Utility of Vitamin D in Acute Coronary Syndrome Patients in Coastal Norway

**DOI:** 10.1155/2015/283178

**Published:** 2015-02-05

**Authors:** Patrycja A. Naesgaard, Volker Pönitz, Hildegunn Aarsetoey, Trygve Brügger-Andersen, Heidi Grundt, William S. Harris, Harry Staines, Dennis W. T. Nilsen

**Affiliations:** ^1^Department of Cardiology, Stavanger University Hospital, Stavanger, Norway; ^2^Department of Clinical Science, University of Bergen, Bergen, Norway; ^3^Department of Medicine, Stavanger University Hospital, Stavanger, Norway; ^4^Department of Medicine, Sanford School of Medicine, University of South Dakota and OmegaQuant Analytics (LLC), Sioux Falls, SD, USA; ^5^Sigma Statistical Services, Balmullo, UK

## Abstract

*Background.* An inverse relationship between cardiovascular risk and levels of vitamin D and omega-3 index may exist.* Objectives.* To evaluate the prognostic utility of serum 25-hydroxyvitamin D [25(OH)D] in 871 patients with suspected acute coronary syndrome (ACS) and to assess the seasonal correlation between 25(OH)D and the omega-3 index in 456 ACS patients from southwestern Norway.* Results.* In the univariate analysis the hazard ratio (HR) at 2-year follow-up for all-cause mortality in the highest as compared to the lowest quartile of 25(OH)D in the total population was 0.61 (95% confidence interval (CI), 0.37–1.00), *P* = 0.050. At 7-year follow-up, the corresponding HR for all-cause mortality was 0.66 (95% CI, 0.49–0.90), *P* = 0.008, and for females alone 0.51 (95% CI, 0.32–0.83), *P* = 0.006. Quartile survival did not differ in the multivariable analysis, whereas 25(OH)D < 40 nM (<16 ng/mL) was found to be independently related to mortality. Seasonal differences in 25(OH)D, but not for the omega-3 index, were noted, and the two biomarkers were positively correlated, especially during winter-spring; Pearson's correlation coefficient was 0.358, *P* < 0.001.* Conclusion.* Vitamin D levels are related to survival, especially in females, and correlate with the omega-3 index.

## 1. Introduction

In observational studies vitamin D and n-3 polyunsaturated fatty acids (PUFA) have been found to be inversely related to cardiovascular disease (CVD) [1, 2]. Whether these factors are causal or are simply markers of a generally lower-risk lifestyle is still unsettled.

Low vitamin D levels have been associated with hypertension, obesity, dyslipidemia, and diabetes mellitus, reflecting an increased cardiovascular (CV) risk burden [3]. However, also high levels can be associated with increased total and CV mortality, as described by a U-shape or reverse J-curve [4–6].

N-3 fatty acids may exert beneficial effects on hemodynamics, lipid metabolism, inflammation, thrombosis, and ventricular arrhythmias [7]. Fish is the major food source of long-chain n-3 PUFA and includes eicosapentaenoic acid (EPA) and docosahexaenoic acid (DHA) [8]. The omega-3 index is a measure of these fatty acids, given as a percentage of total fatty acids in red blood cell membranes [9].

Vitamin D is mainly synthesized in the skin from 7-dehydrocholesterol under the influence of sun exposure and the remaining 10–20% is ingested in the diet in which fatty fish is a major contributor [10]. The synthesis of vitamin D in response to ultraviolet B (UVB) radiation in sunlight depends on factors such as latitude, altitude, time of year and day-time, weather, age, skin pigmentation type, clothing, and sunscreen and is influenced by environment (pollution) and lifestyle [11]. Conditions such as poor nutrition and chronic illness are also associated with vitamin D deficiency [12].

The prognostic utility of vitamin D in relation to long-term CV outcomes has been evaluated in subjects without a history of CVD, as in the Framingham Offspring study [13] and in patients with established CVD [14, 15]. We have previously shown that 2- and 5-year total and cardiac mortality is decreased in the highest as compared to the lowest quartile of vitamin D in a coronary chest-pain population living at altitudes exceeding 1000 meters in subtropical northern Argentina [16], and this relationship is stronger in females than in males [17].

The main aim of our study was to evaluate the prognostic utility of vitamin D measured as serum 25-hydroxyvitamin D [25(OH)D] in a Norwegian coastal coronary chest-pain population exposed to temperate sun activity at low altitudes and with a high consumption of fish as reflected by the omega-3 index. As a secondary aim, we looked into the seasonal correlation between 25(OH)D and the omega-3 index in the subjects with troponin-T (TnT) positive values.

## 2. Methods

Participants in the present analysis belonged to the RACS study (Risk Markers in the Acute Coronary Syndrome) (ClinicalTrials.gov NCT00521976), a single-center prospective observational study designed to evaluate the prognostic utility of serum 25(OH)D (D represents D_2_ and D_3_) status in 871 patients with chest pain and suspected ACS consecutively admitted to the Stavanger University Hospital from November 2002 to October 2003. The details of the RACS study have been published previously [18]. The primary outcome in the present study was all-cause mortality at 2- and 7-year follow-up and the secondary outcomes included cardiac death and sudden cardiac death (SCD) at 2-year follow-up, as previously defined [16]. Measurements of the omega-3 index at admission were previously investigated in 456 of the 471 ACS patients (defined by a peak baseline TnT value > 0.01 ng/mL) [19]. In this subpopulation we performed a seasonal (summer-autumn defined as June–November and winter-spring defined as December–May) correlation between levels of 25(OH)D and the omega-3 index. 25(OH)D as a prognostic marker was evaluated in the total population, as well as in subgroups including TnT positive and TnT negative patients, gender, and vitamin D deficient subjects (defined as 25(OH)D < 40 nM (16 ng/mL)) [20]. Furthermore, seasonal variations in 25(OH)D were evaluated in the total population. Data were analyzed in quartiles of 25(OH)D and as continuous 25(OH)D values applying univariate and multivariable analyses.

The study was approved by the Regional Board of Research Ethics and the Norwegian Health Authorities and conducted in accordance with the Helsinki Declaration of 1971, as revised in 1983. Written informed consent was obtained from all patients.

Blood samples were drawn immediately following admission by direct venipuncture of an antecubital vein, applying a minimum of stasis. A second blood sample for TnT determination was drawn six hours following the first sample. Baseline laboratory data for this study included measurements of 25(OH)D_2_, 25(OH)D_3_, TnT, high sensitivity C-reactive protein (hsCRP), glucose, serum lipids, B-type natriuretic peptide (BNP) measured in EDTA (ethylene diamine tetraacetic acid) plasma, estimated glomerular filtration rate (eGFR) (calculated by modification in diet in renal disease (MDRD) formula), and omega-3 index.

TnT was quantified by a cardiac-specific second-generation troponin T ELISA assay from Roche Diagnostics, using a high-affinity cardiac-specific TnT isoform antibody [21]. The lower detection limit of the assay used is 0.01 ng/mL.

25(OH)D analysis was carried out at the Department of Medical Biochemistry at Stavanger University Hospital, as previously described [16].

The omega-3 index (EPA + DEA) content was measured in packed red blood cells, as previously described by Harris and von Schacky [9] and by Aarsetoey et al. [19].

### 2.1. Statistical Analysis

Patients were grouped into quartiles according to their 25(OH)D levels within the total population and TnT positive and negative subpopulations as appropriate. As a sensitivity analysis, selected analyses were conducted using continuous values instead of quartiles. Approximately normally distributed variables were given as mean and standard deviation (SD). The Chi-square test for association was applied between the 25(OH)D quartiles and categorical variables at baseline. The one way analysis of variance (ANOVA) was used to test for equality of means of scale variables (e.g., age) amongst 25(OH)D quartiles. The hazard ratios (HR) are presented with 95% confidence interval (CI). Separate stepwise Cox multivariable proportional hazards regression models with total death, cardiac death and SCD as the dependent variable, and 25(OH)D and other variables as potential independent predictors (listed below) were fitted. To examine the differences in prognosis between subjects in the upper quartile(s) versus the lowest quartile of 25(OH)D, we adjusted for gender, age, smoking, hypertension, index diagnosis, diabetes mellitus, congestive heart failure (CHF) (defined by Killip-Kimball class at admission; patients in class 2 to 4 were classified as CHF patients and those in class 1 as non-CHF), history of previous coronary heart disease (CHD) (i.e., history of either angina pectoris, myocardial infarction, percutaneous coronary intervention, or coronary artery bypass graft), hypercholesterolemia/use of statins, TnT > 0.01 ng/mL, eGFR, hsCRP, BNP, body mass index (kg/m^2^), and beta-blockers prior to enrolment. The Kaplan-Meier product limits were used for plotting times to event with the equality of the 25(OH)D quartile survival curves assessed by the log-rank test. Hypothesis two-sided *P* values less than 0.05 are defined as statistically significant.

## 3. Results 

A total of 871 patients (531 men and 340 women) were enrolled in the RACS study. Ten samples were not available; thus 861 patients (525 men and 336 women) were included in the present 25(OH)D analysis. Of these, 467 patients (54%) (305 men and 162 women) were characterized by a TnT release > 0.01 ng/mL. Mean age in the total patient population was 69.6 ± 14.4 years (males 66.6 ± 14.3 years and females 74.4 ± 13.1 years). Mean 25(OH)D levels were 52.7 ± 19.1 nM (21.1 ± 7.6 ng/mL) in the total patient population; males 53.8 ± 19.3 nM (21.5 ± 7.7 ng/mL) and females 51.0 ± 18.8 nM (20.4 ± 7.5 ng/mL). Seasonal 25(OH)D levels in all patients were 51.6 ± 18.9 nM (20.6 ± 7.6 ng/mL) during winter-spring and 55.4 ± 19.1 nM (22.2 ± 7.6 ng/mL) during summer-autumn; *P* = 0.007. The seasonal difference was greatest in males (52.1 nM ± 19.1 nM versus 57.7 nM ± 19.1 nM; *P* = 0.002) and not significant in women (50.7 nM ± 18.5 nM versus 51.6 nM ± 19.5 nM; *P* = 0.711).

The baseline characteristics according to 25(OH)D quartiles at admission in the total patient population, in the TnT positive group, and in both genders are shown in Tables 1, 2, and 3(a)-3(b), respectively.

### 3.1. 2-Year Follow-Up

At 2-year follow-up, 136 (70 men and 66 women) patients with known 25(OH)D values had died, 84 (62%) of whom died due to a cardiac event, and of these, 25 cases were defined as SCD. In the TnT positive group 104 patients with known 25(OH)D values died: 71 (68%) due to a cardiac event, including 21 sudden cardiac deaths.

In the univariate quartile analysis of all-cause mortality in the total population and in the TnT positive patient population, the HRs for 25(OH)D in the highest quartile (Q4) as compared to the lowest quartile (Q1) were 0.61 (95% CI, 0.37–1.00), *P* = 0.050, and 0.59 (95% CI, 0.33–1.04), *P* = 0.067, respectively. No difference in HR was found between 25(OH)D quartiles in the TnT negative population, nor for cardiac death in the total and TnT positive population. The results remained statistically nonsignificant in the multivariable analysis for all groups. The quartile univariate and multivariable results are presented in Table 4.

In the univariate analysis of continuous 25(OH)D values in the total population, the HRs for total mortality and cardiac death were 0.99 (95% CI, 0.98–1.00), *P* = 0.035, and 0.99 (95% CI, 0.98–1.00), *P* = 0.060, respectively. In the univariate analysis of the TnT positive population, the HRs for total mortality and cardiac death were 0.99 (95% CI, 0.99–1.00), *P* = 0.012, and 0.99 (95% CI, 0.98–1.00), *P* = 0.055, respectively. The remaining continuous univariate and multivariable results are shown in Table 5.

### 3.2. 2-Year Gender Analysis

In the univariate quartile analysis of total and cardiac mortality in the female population, the HRs for 25(OH)D in Q4 as compared to Q1 were 0.43 (95% CI, 0.19–0.94), *P* = 0.035, and 0.35 (95% CI, 0.13–0.98), *P* = 0.046, respectively. No difference in the HR was found between the 25(OH)D quartiles in males. The 25(OH)D quartiles' HRs for SCD were not significant in either the univariate or multivariable models. The quartile univariate and multivariable results are presented in Table 4.

In the univariate analysis of continuous 25(OH)D values in the female population, the HRs for total mortality and cardiac death were 0.98 (95% CI, 0.97–1.00), *P* = 0.023, and 0.98 (95% CI, 0.97–1.00), *P* = 0.044, respectively. The continuous univariate and multivariable results are presented in Table 5.

### 3.3. 7-Year Follow-Up

**Figure 1:**
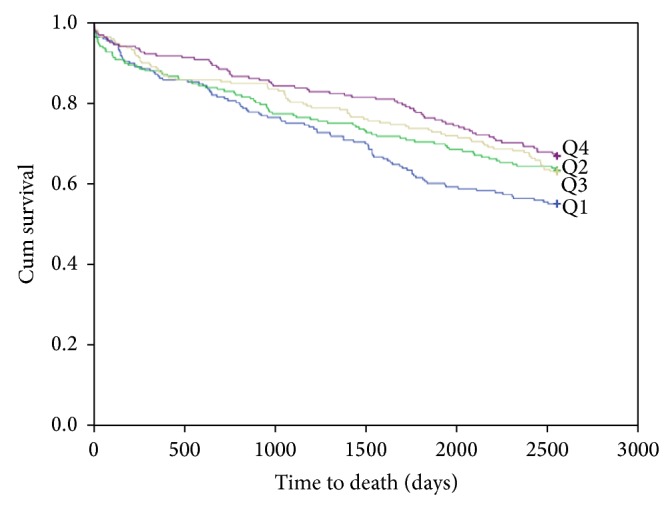
Kaplan-Meier plots for seven-year total mortality of 25(OH)D quartiles in the total patient population.

**Figure 2:**
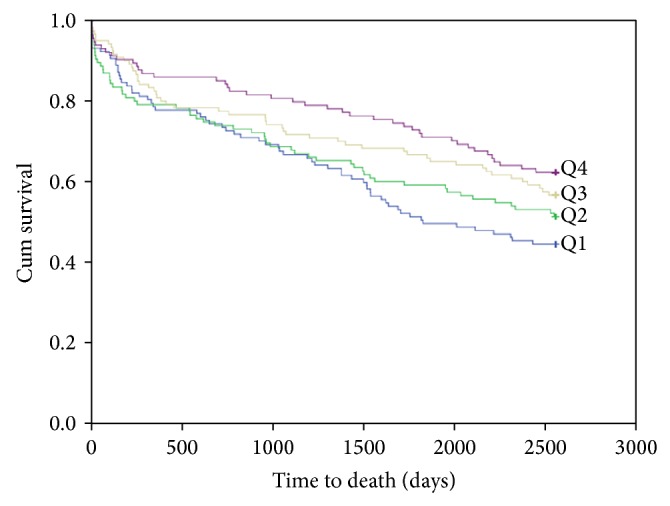
Kaplan-Meier plots for seven-year total mortality of 25(OH)D quartiles in the TnT positive patient population.

At 7-year follow-up, data collection was restricted to total mortality. During this period 327 (180 men and 147 women) patients had died. The Kaplan-Meier curve for survival is shown in Figure 1. In the TnT positive group, 216 patients (46%) died, as shown in Figure 2.

In the univariate quartile analysis of all-cause mortality in the total population and in the TnT positive patient population, the HRs for 25(OH)D in Q4 as compared to Q1 were 0.66 (95% CI, 0.48–0.89), *P* = 0.007, and 0.60 (95% CI, 0.41–0.87), *P* = 0.008, respectively. No difference in mortality was found amongst the 25(OH)D quartiles in the TnT negative population. The results were statistically nonsignificant in the multivariable analysis for all groups. The quartile univariate and multivariable results are presented in Table 4.

In the univariate analysis of continuous 25(OH)D values in the total population and in the TnT positive patient population, the HRs for total mortality were 0.99 (95% CI, 0.99–1.00), *P* = 0.026, and 0.99 (95% CI, 0.98–1.00), *P* = 0.002, respectively. The continuous univariate and multivariable results are presented in Table 5.

Evaluating the influence of diabetes in the total population, the univariate analysis at 7-year follow-up revealed a significant difference in total mortality, *P* < 0.001, not found in the multivariable analysis.

### 3.4. 7-Year Gender Analysis

**Figure 3:**
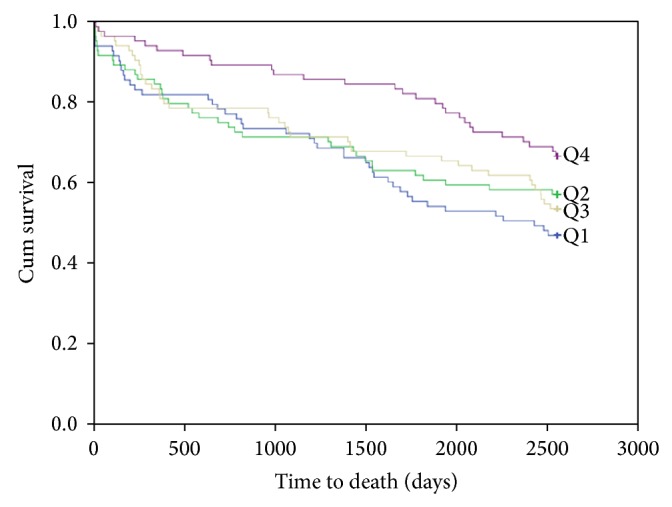
Kaplan-Meier plots for seven-year total mortality of 25(OH)D quartiles in females.

The univariate quartile analysis showed a statistically significant difference in the HR of Q4 as compared to Q1 of 25(OH)D for total death among females 0.51 (95% CI, 0.32–0.83), *P* = 0.006, but not amongst males. The KM curve for survival in females is shown in Figure 3. The difference found in women was no longer significant in the multivariable analysis. The quartile univariate and multivariable results are presented in Table 4.

In the univariate analysis of continuous 25(OH)D values in the female population, the HR for total mortality was 0.99 (95% CI 0.98–1.00), *P* = 0.025. The continuous univariate and multivariable results are presented in Table 5.

### 3.5. 7-Year Follow-Up of Vitamin D Deficient [<40 nM (<16 ng/mL)] Patients

There were 232 patients (27%) with 25(OH)D levels below 40 nM (16 ng/mL) and 629 patients (73%) with 25(OH)D levels above and equal to 40 nM (16 ng/mL). The univariate HR for total mortality in patients with 25(OH)D deficiency was 1.43 (95% CI, 1.14–1.81); *P* = 0.002, as compared to subjects without 25(OH)D deficiency and was still significant in the multivariable model; HR 1.32 (95% CI, 1.04–1.68), *P* = 0.024.

### 3.6. Seasonal Correlation between Vitamin D and Omega-3 Index

456 patients (298 men and 158 women) were included in the seasonal correlation study between 25(OH)D and omega-3 index. Mean 25(OH)D levels in this subgroup were 50.4 ± 18.2 nM (29.2 ± 7.3 ng/mL) winter-spring and 55.2 ± 20.1 nM (22.1 ± 8.0 mg/mL) during the summer-autumn season; *P* = 0.009. Corresponding levels of the omega-3 index were 6.8(1.9)% and 6.6(1.9)%, respectively; *P* = 0.26. In this subgroup, there was a strong positive correlation between 25(OH)D and the omega-3 index during both seasons. For the total population the Pearson's correlation coefficient was 0.358, *P* < 0.001, during the winter-spring season and 0.199, *P* = 0.006, during the summer-autumn period, respectively. The Pearson's seasonal correlation coefficient for females was 0.380, *P* < 0.001 and 0.183 *P* = 0.16, respectively, and for males 0.347, *P* < 0.001 and 0.205, *P* = 0.022, respectively.

## 4. Discussion

Our chest pain population was recruited from the southwestern coast of Norway, at a latitude of 58°N. At this latitude, the production of cutaneous vitamin D is low in the winter months during which vitamin D levels depend on other sources, mainly fish intake.

We divided the population into quartiles of vitamin D levels, comparing the higher quartiles to the lowest quartile, and performed univariate and multivariable analyses of mortality at 2- and 7-year follow-up.

In the univariate quartile analysis at 2-year follow-up of the total and TnT positive patient population we observed a trend towards decreased total and cardiac mortality in the upper as compared to the lower quartile of vitamin D. These trends approached significant values when applying continuous vitamin D data in the univariate analysis (Table 5). In the female population, the reduction in total and cardiac mortality reached statistical significance by both quartile and continuous univariate analysis.

At 7-year follow-up, both statistical approaches yielded significant results in the univariate analysis, with reduced total and cardiac mortality associated with increasing vitamin D levels in the total and TnT positive population, as well as in the female population, respectively. These and other 7-year follow-up data are shown in Table 5.

These associations were not present after adjusting for confounding variables in our multivariable model. In contrast to studies in which multivariable models have demonstrated a U-shape or reverse J-shape mortality curve across quintiles [4] and quartiles [5] of vitamin D, the statistically nonsignificant results in our multivariable quartile analysis precluded a similar evaluation. However, the univariate quartile hazard ratios in our ACS population showed a similar pattern to that of the above studies [4, 5], in which subjects were enrolled from a general elderly population, with the highest age (>75 years) in the study by Jia et al. [4] as compared to 69.6 ± 14.4 years in our study. In general, the vitamin D values in our patients were higher than in the two other studies, with the lowest quartile in our study comparable to that of second and third quintiles in the study by Jia et al. [4] and to the second quartile in the study by Visser et al. [5]. Furthermore, less interquartile differences in morbidity and mortality in our study may be related to a tighter range of vitamin D values.

In a previous report from our group we obtained more definitive results investigating a population in Northern Argentina located at a latitude of 24° and at an altitude more than 1000 m above sea level. In the latter population, the prognostic utility of vitamin D in the female population remained highly significant after correcting for confounding factors, which statistically may be due to a larger proportion of females and more events in this subgroup. Similar results have been obtained in a German study by Karakas et al. [22]. However, in that study they included 1783 healthy middle-aged subjects of both gender with a follow-up of eleven years during which they identified 298 combined CHD cases, whereas we investigated total and cardiac death in chest pain patients with suspected ACS.

In our Norwegian as compared to our Argentinean study [16] levels of vitamin D were similar in the lowest quartile (30.5 ± 5.9 nM versus 30.7 ± 5.6 nM) of the total populations. Levels were also similar in Q2 and Q3, but higher in Q4 in the Norwegian population (78.6 ± 13.2 nM versus 72.9 ± 11.1 nM). This was an unexpected finding, as the geographic locations are different with respect to sun exposure.

We have previously [23] published data suggesting that vitamin D status may be related to socioeconomic factors, implying that low vitamin D levels in that setting may behave as a secondary risk marker. In our present population the quartile levels of vitamin D were clearly related to the difference in dietary capture of vitamin D, as demonstrated by a monotonic increase in omega-3 values from lowest to highest quartile of vitamin D, as shown in Table 2.

Based on our observational data, reduced vitamin D levels may not only be regarded as a marker of morbidity in women as compared to men but may also reflect a gender difference in serum vitamin D concentration. The mechanism for this difference is not obvious, although a female hormonal interaction has been noted, with levels of vitamin D and progesterone/estrogen being inversely correlated [24].

Optimal levels of 25(OH)D are still under debate. According to Ross et al. [20], levels of at least 50 nM will meet the needs of at least 97.5% of the population, whereas the median vitamin D concentration in the general population will be above 40 nM. In the present study, we have investigated the significance of vitamin D levels < 40 nM in relation to outcome. Below 40 nM (16 ng/mL), vitamin D behaved as an independent predictor for total death both in the univariate and multivariable analysis. As vitamin D deficiency was found to be an independent risk factor at levels below 40 nM, higher levels may reflect an overall healthier lifestyle and may not in themselves be causally related to outcome.

In a subpopulation consisting of TnT positive patients, the correlation between vitamin D and the omega-3 index, including EPA and DHA, was higher during winter-spring (*r* = 0.358, *P* < 0.001) as compared to the summer-autumn season (*r* = 0.199, *P* = 0.006). As the omega-3 index was similar in both seasons, this finding may be explained by an increased utilization of vitamin D in the diet during the winter season, and the lower correlation during the summer season may be due to the increased levels of cutaneous vitamin D production. Our results are in accordance with a recent publication in which the authors compared levels of vitamin D obtained from sun exposure versus diet at the same altitude as in the present study [25].

The association between vitamin D and omega-3, especially during the winter-spring season, is of interest, as the diet may be the main source of vitamin D during this period. Furthermore, the correlation was strongest in women in whom vitamin D showed less seasonal variation. Thus, due to its prognostic implication, vitamin D may be involved in the beneficial effects of fish intake. Therefore, the natural presence of vitamin D in dietary studies, such as in DART [26] and in the long-term follow-up study by Kromhout et al. [27], may have beneficially affected outcome in these studies, as reflected by the stepwise increase in the omega-3 index from lowest to highest quartile of vitamin D in the present study. Although reduction in mortality was noted in the omega-3 arm of the GISSI studies [28, 29], some of the other megatrials also applying a purified omega-3 compound have not shown a reduction in mortality, as noted in a meta-analysis by Kwak et al. [30], involving 20485 patients with a history of cardiovascular disease from 14 randomized, double blind, and placebo controlled studies.

The addition of vitamin D to a purified omega-3 compound is being investigated in the VITAL study (ClinicalTrials.gov Identifier: NCT01169259).

*Strengths and Limitations*. In comparison to other observational studies enrolling subjects from a general population, the novelty in this study is related to (1) selection of a chest pain population with suspected ACS, (2) blood samples obtained at hospital admission, and (3) categorization of subjects with and without myocardial injury. In contrast to many other studies, we have focused not only on total mortality but also on cardiac death in our selected high risk population. The strength of our study is mainly related to the long-term follow-up and the proportion of females accounting for 39% of the patients. Furthermore, its design is similar to that of our previously published Argentinean report based on admitted chest pain patients with suspected ACS [16], enabling a comparison of western lifestyle and traditions to that of a South-American population with other traditions and dietary habits. A larger number of patients may have contributed to more consistent findings in our Argentinean population. However, as power calculations are not applicable in observational studies, this should be regarded as a limitation. Also, we did not correct for parathyroid hormone (PTH), but we do not regard this to be of great importance, as previous data would indicate that only very low values of vitamin D will influence the level of PTH [31]. As this is an observational study, unknown confounders may have been missed.

In conclusion, at long-term follow-up the univariate analysis demonstrated a statistically significant lower mortality in the highest as compared to the lowest quartile of vitamin D in chest pain patients with suspected ACS, especially in females. Vitamin D was independently related to mortality at levels below the population median. An increase in the omega-3 index was noted from lowest to highest quartile of vitamin D and there was a stronger correlation between vitamin D and the omega-3 index during winter-spring as compared to the summer-autumn season.

## Figures and Tables

**Table 1:** Baseline characteristics of the total population arranged according to the quartiles of 25(OH)D.

Characteristics *n* (%)	Quartiles of 25(OH)D				*P* value
	Q1	Q2	Q3	Q4	
25(OH)D (nmol/L)^*^	30.5 ± 5.9	45.2 ± 3.4	56.7 ± 3.6	78.6 ± 13.2	<0.001
25(OH)D (ng/mL)^*^	12.2 ± 2.4	18.1 ± 1.4	22.7 ± 1.4	31.4 ± 5.3	<0.001
Age, years^*^	68.9 ± 16.1	69.4 ± 15.6	70.7 ± 13.2	69.3 ± 12.4	0.591
Male, *n* (%)	120 (55.8)	138 (64.2)	127 (58.8)	140 (65.1)	0.150
Smoking status, *n* (%)					0.110
Current smoker, *n* (%)	71 (33.0)	58 (27.0)	46 (21.3)	52 (24.2)	
Past smoker, *n* (%)	65 (30.2)	78 (36.3)	83 (38.4)	87 (40.5)	
Never smoked, *n* (%)	79 (36.7)	79 (36.7)	87 (40.3)	76 (35.3)	
Angina pectoris, *n* (%)	93 (43.3)	90 (41.9)	100 (46.3)	93 (43.3)	0.820
CHF, *n* (%)					
Killip Class 2–4	84 (39.1)	48 (22.3)	52 (24.1)	48 (22.3)	<0.001
History of previous MI, *n* (%)	82 (38.1)	69 (32.1)	71 (32.9)	66 (30.7)	0.382
CABG, *n* (%)	22 (10.2)	17 (7.9)	26 (12.0)	22 (10.2)	0.564
PCI, *n* (%)	18 (8.4)	21 (9.8)	25 (11.6)	23 (10.7)	0.721
Hypertension, *n* (%)	87 (40.5)	86 (40.0)	96 (44.4)	93 (43.3)	0.746
History of DM 1, *n* (%)	2 (0.9)	2 (0.9)	0 (0.0)	4 (1.9)	0.256
History of DM 2, *n* (%)	38 (17.7)	24 (11.2)	34 (15.7)	15 (7.0)	0.004
STEMI, *n* (%)	30 (14.0)	35 (16.3)	31 (14.4)	32 (14.9)	0.913
TnT release, *n* (%)	117 (54.4)	115 (53.5)	120 (55.6)	115 (53.5)	0.969
eGFR (*μ*mol L^−1^)^*^	63.5 ± 22.1	61.7 ± 20.8	62.9 ± 20.2	63.0 ± 20.3	0.834
Cholesterol/Statin, *n* (%)	104 (48.4)	105 (48.8)	109 (50.5)	107 (49.8)	0.973
Beta-blocker, *n* (%)	81 (37.7)	78 (36.3)	77 (35.6)	73 (34.0)	0.881
Known CHD, *n* (%)	140 (65.1)	132 (61.4)	142 (65.7)	129 (60.0)	0.537
BMI (kg/m^2^)^*^	25.7 ± 4.4	26.3 ± 4.7	25.7 ± 4.2	25.0 ± 3.7	0.024
BNP quartiles					0.222
Q1	47 (22.8)	52 (24.8)	44 (21.4)	64 (31.7)	
Q2	50 (24.3)	50 (23.8)	60 (29.1)	44 (21.8)	
Q3	48 (23.3)	53 (25.2)	54 (26.2)	53 (26.2)	
Q4	61 (29.6)	55 (26.2)	48 (23.3)	41 (20.3)	
hsCRP quartiles					0.910
Q1	51 (23.7)	52 (24.2)	53 (24.5)	61 (28.4)	
Q2	49 (22.8)	50 (23.3)	56 (25.9)	57 (26.5)	
Q3	57 (26.5)	57 (26.5)	53 (24.5)	50 (23.3)	
Q4	58 (27.0)	56 (26.0)	54 (25.0)	47 (21.9)	

^*^Mean ± SD.

SD, standard deviation; 25(OH)D, 25-hydroxyvitamin D; CHF, congestive heart failure; MI, myocardial infarction; CABG, coronary artery bypass grafting; PCI, percutaneous coronary intervention; DM, diabetes mellitus; STEMI, ST-elevation myocardial infarction; TnT, troponin T; eGFR, estimated glomerular filtration rate; CHD, coronary heart disease; BMI, body mass index; BNP, B-type natriuretic peptide; hsCRP, high sensitivity C-reactive protein.

**Table 2:** Baseline characteristics of the TnT positive patient population arranged according to the quartiles of 25(OH)D.

Characteristics *n* (%)	Quartiles of 25(OH)D				*P* value
	Q1	Q2	Q3	Q4	
25(OH)D (nmol/L)^*^	30.2 ± 6.2	44.9 ± 3.5	56.6 ± 3.6	78.1 ± 13.2	<0.001
25(OH)D (ng/mL)^*^	12.1 ± 2.5	18.0 ± 1.4	22.6 ± 1.4	31.2 ± 5.3	<0.001
Omega-3 Index^*^	6.1 ± 1.6	6.4 ± 1.6	6.9 ± 1.9	7.5 ± 2.2	<0.001
Age, years^*^	72.5 ± 14.8	72.6 ± 13.5	71.5 ± 13.8	71.2 ± 11.0	0.809
Male, *n* (%)	64 (55.2)	81 (69.2)	73 (62.4)	86 (74.1)	0.015
Smoking status, *n* (%)					0.047
Current smoker, *n* (%)	36 (31.0)	37 (31.6)	35 (29.9)	30 (25.9)	
Past smoker, *n* (%)	30 (25.9)	42 (35.9)	35 (29.9)	53 (45.7)	
Never smoked, *n* (%)	50 (43.1)	38 (32.5)	47 (40.2)	33 (28.4)	
Angina pectoris, *n* (%)	49 (42.2)	48 (41.0)	52 (44.4)	51 (44.0)	0.949
CHF, *n* (%)					
Killip Class 2–4	56 (48.3)	27 (23.1)	30 (25.6)	32 (27.6)	<0.001
History of previous MI, *n* (%)	46 (39.7)	37 (31.6)	35 (29.9)	38 (32.8)	0.413
CABG, *n* (%)	11 (9.5)	7 (6.0)	12 (10.3)	12 (10.3)	0.612
PCI, *n* (%)	9 (7.8)	9 (7.7)	7 (6.0)	10 (8.6)	0.893
Hypertension, *n* (%)	48 (41.4)	47 (40.2)	52 (44.4)	49 (42.2)	0.926
History of DM 1, *n* (%)	0 (0.0)	2 (1.7)	0 (0.0)	4 (3.4)	0.058
History of DM 2, *n* (%)	26 (22.4)	19 (16.2)	19 (16.2)	9 (7.8)	0.023
STEMI, *n* (%)	29 (25.0)	35 (29.9)	30 (25.6)	33 (28.4)	0.813
eGFR (*μ*mol L^−1^)^*^	59.7 ± 23.2	59.8 ± 22.3	62.5 ± 23.3	63.9 ± 23.5	0.418
Cholesterol/statin, *n* (%)	53 (45.7)	54 (46.2)	52 (44.4)	50 (43.1)	0.967
Beta-blocker, *n* (%)	40 (34.5)	39 (33.3)	35 (29.9)	34 (29.3)	0.793
Known CHD, *n* (%)	80 (69.0)	71 (60.7)	76 (65.0)	72 (62.1)	0.566
BMI (kg/m^2^)^*^	25.7 ± 4.5	25.9 ± 4.6	25.6 ± 4.3	25.3 ± 3.9	0.714
BNP quartiles					0.778
Q1	15 (13.8)	17 (15.0)	22 (19.5)	23 (21.3)	
Q2	24 (22.0)	23 (20.4)	25 (22.1)	24 (22.2)	
Q3	26 (23.9)	34 (30.1)	32 (28.3)	26 (24.1)	
Q4	44 (40.4)	39 (34.5)	34 (30.1)	35 (32.4)	
hsCRP quartiles					0.698
Q1	18 (15.5)	22 (18.8)	19 (16.2)	27 (23.3)	
Q2	24 (20.7)	27 (23.1)	31 (26.5)	27 (23.3)	
Q3	32 (27.6)	36 (30.8)	36 (30.8)	32 (27.6)	
Q4	42 (36.2)	32 (27.4)	31 (26.5)	30 (25.9)	

^*^Mean ± SD.

SD, standard deviation; 25(OH)D, 25-hydroxyvitamin D; CHF, congestive heart failure; MI, myocardial infarction; CABG, coronary artery bypass grafting; PCI, percutaneous coronary intervention; DM, diabetes mellitus; STEMI, ST-elevation myocardial infarction; TnT, troponin T; eGFR, estimated glomerular filtration rate; CHD, coronary heart disease; BMI, body mass index; BNP, B-type natriuretic peptide; hsCRP, high sensitivity C-reactive protein.

**(a):** 

Characteristics *n* (%)	Quartiles of 25(OH)D				*P* value
	Q1	Q2	Q3	Q4	
25(OH)D (nmol/L)^*^	28.8 ± 5.6	43.2 ± 3.9	55.6 ± 3.4	76.1 ± 12.3	<0.001
25(OH)D (ng/mL)^*^	11.5 ± 2.2	17.3 ± 1.6	22.2 ± 1.4	30.4 ± 4.9	<0.001
Age, years^*^	75.5 ± 14.3	75.8 ± 12.9	75.0 ± 12.1	70.9 ± 12.8	0.058
Smoking status, *n* (%)					0.384
Current smoker, *n* (%)	18 (21.7)	14 (16.7)	10 (11.9)	17 (20.2)	
Past smoker, *n* (%)	13 (15.7)	21 (25.0)	17 (20.2)	21 (25.0)	
Never smoked, *n* (%)	52 (62.7)	49 (58.3)	57 (67.9)	46 (54.8)	
Angina pectoris, *n* (%)	44 (53.0)	45 (53.6)	39 (46.3)	39 (46.4)	0.663
CHF, *n* (%)					
Killip class 2–4	43 (51.8)	26 (31.0)	20 (23.8)	17 (20.2)	<0.001
History of previous MI, *n* (%)	30 (36.1)	25 (29.8)	25 (29.8)	22 (26.2)	0.567
CABG, *n* (%)	6 (7.2)	3 (3.6)	8 (9.5)	3 (3.6)	0.279
PCI, *n* (%)	1 (1.2)	2 (2.4)	7 (8.3)	8 (9.5)	0.035
Hypertension, *n* (%)	41 (49.4)	39 (46.4)	40 (47.6)	37 (44.4)	0.917
History of DM 1, *n* (%)	1 (1.2)	1 (1.2)	0 (0.0)	1 (1.2)	0.798
History of DM 2, *n* (%)	11 (13.3)	12 (14.3)	13 (15.5)	9 (10.7)	0.828
STEMI, *n* (%)	11 (13.3)	7 (8.3)	3 (3.6)	7 (8.3)	0.164
TnT release, *n* (%)	46 (55.4)	40 (47.6)	42 (50.0)	34 (40.5)	0.277
eGFR (*μ*mol L^−1^)^*^	52.3 ± 18.4	54.9 ± 17.4	53.9 ± 17.8	58.9 ± 15.1	0.079
Cholesterol/statin, *n* (%)	43 (51.8)	46 (54.8)	45 (53.6)	45 (53.6)	0.985
Beta-blocker, *n* (%)	33 (39.8)	32 (38.1)	38 (45.2)	23 (27.4)	0.112
Known CHD, *n* (%)	59 (71.1)	56 (66.7)	56 (66.7)	50 (59.5)	0.465
BMI (kg/m^2^)^*^	25.4 ± 4.8	25.5 ± 4.6	25.2 ± 5.2	24.9 ± 4.0	0.886
BNP quartiles					0.094
Q1	17 (20.7)	20 (25.0)	17 (21.5)	27 (33.8)	
Q2	19 (23.2)	23 (28.8)	20 (25.3)	19 (23.8)	
Q3	15 (18.3)	21 (26.3)	23 (29.1)	21 (26.3)	
Q4	31 (37.8)	16 (20.0)	19 (24.1)	13 (16.3)	
hsCRP quartiles					0.995
Q1	20 (24.1)	19 (22.6)	21 (25.0)	24 (28.6)	
Q2	21 (25.3)	20 (23.8)	22 (26.2)	20 (23.8)	
Q3	21 (25.3)	22 (26.2)	19 (22.6)	22 (26.2)	
Q4	21 (25.3)	23 (27.4)	22 (26.2)	18 (21.4)	

^*^Mean ± SD.

SD, standard deviation; 25(OH)D, 25-hydroxyvitamin D; CHF, congestive heart failure; MI, myocardial infarction; CABG, coronary artery bypass grafting; PCI, percutaneous coronary intervention; DM, diabetes mellitus; STEMI, ST-elevation myocardial infarction; TnT, troponin T; eGFR, estimated glomerular filtration rate; CHD, coronary heart disease; BMI, body mass index; BNP, B-type natriuretic peptide; hsCRP, high sensitivity C-reactive protein.

**(b):** 

Characteristics *n* (%)	Quartiles of 25(OH)D				*P* value
	Q1	Q2	Q3	Q4	
25(OH)D (nmol/L)^*^	31.7 ± 6.0	46.3 ± 3.0	57.4 ± 3.8	79.9 ± 13.7	<0.001
25(OH)D (ng/mL)^*^	12.7 ± 2.4	18.5 ± 1.2	23.0 ± 1.5	32.0 ± 5.5	<0.001
Age, years^*^	63.6 ± 15.3	66.0 ± 16.3	67.8 ± 12.9	68.6 ± 12.3	0.024
Smoking status, *n* (%)					0.211
Current smoker, *n* (%)	53 (40.5)	44 (33.6)	37 (28.0)	34 (26.0)	
Past smoker, *n* (%)	53 (40.5)	58 (44.3)	67 (50.8)	63 (48.1)	
Never smoked, *n* (%)	25 (19.1)	29 (22.1)	28 (21.2)	34 (26.0)	
Angina pectoris, *n* (%)	46 (35.1)	48 (36.6)	60 (45.5)	54 (41.2)	0.306
CHF, *n* (%)					
Killip Class 2–4	42 (32.1)	21 (16.0)	32 (24.2)	31 (23.7)	0.026
History of previous MI, *n* (%)	50 (38.2)	46 (35.1)	45 (34.1)	44 (33.6)	0.867
CABG, *n* (%)	18 (13.7)	14 (10.7)	16 (12.1)	19 (14.5)	0.795
PCI, *n* (%)	19 (14.5)	17 (13.0)	18 (13.6)	15 (11.5)	0.903
Hypertension, *n* (%)	44 (33.6)	45 (34.4)	56 (42.4)	59 (45.0)	0.141
History of DM 1, *n* (%)	1 (0.8)	1 (0.8)	1 (0.8)	2 (1.5)	0.894
History of DM 2, *n* (%)	25 (19.1)	14 (10.7)	19 (14.4)	8 (6.1)	0.013
STEMI, *n* (%)	21 (16.0)	28 (21.4)	28 (21.2)	23 (17.6)	0.610
TnT release, *n* (%)	73 (55.7)	74 (56.5)	76 (57.6)	81 (61.8)	0.754
eGFR (*μ*mol L^−1^)^*^	70.5 ± 21.7	67.0 ± 21.0	68.1 ± 20.6	65.8 ± 21.7	0.312
Cholesterol/statin, *n* (%)	57 (43.5)	62 (47.3)	65 (49.2)	61 (46.6)	0.826
Beta-blocker, *n* (%)	49 (37.4)	44 (33.6)	44 (33.3)	45 (34.4)	0.894
Known CHD, *n* (%)	80 (61.1)	77 (58.8)	85 (64.4)	79 (60.3)	0.817
BMI (kg/m^2^)^*^	26.1 ± 3.9	26.6 ± 4.9	26.0 ± 3.4	25.0 ± 3.5	0.014
BNP quartiles					0.545
Q1	29 (23.4)	35 (27.3)	27 (21.1)	36 (29.5)	
Q2	30 (24.2)	30 (23.4)	39 (30.5)	27 (22.1)	
Q3	30 (24.2)	27 (21.1)	36 (28.1)	31 (25.4)	
Q4	35 (28.2)	36 (28.1)	26 (20.3)	28 (23.0)	
hsCRP Quartiles					0.904
Q1	30 (22.9)	33 (25.2)	32 (24.2)	36 (27.5)	
Q2	28 (21.4)	31 (23.7)	35 (26.5)	36 (27.5)	
Q3	37 (28.2)	34 (26.0)	35 (26.5)	27 (20.6)	
Q4	36 (27.5)	33 (25.2)	30 (22.7)	32 (24.4)	

^*^Mean ± SD.

SD, standard deviation; 25(OH)D, 25-hydroxyvitamin D; CHF, congestive heart failure; MI, myocardial infarction; CABG, coronary artery bypass grafting; PCI, percutaneous coronary intervention; DM, diabetes mellitus; STEMI, ST-elevation myocardial infarction; TnT, troponin T; eGFR, estimated glomerular filtration rate; CHD, coronary heart disease; BMI, body mass index; BNP, B-type natriuretic peptide; hsCRP, high sensitivity C-reactive protein.

**Table 4:** The univariate and multivariable HRs (95% CI) for quartiles of 25(OH)D.

	2-year follow-up		7-year follow-up	
	Univariate analysis	Multivariable analysis^*^	Univariate analysis	Multivariable analysis^*^
Total population				
Total mortality	0.61 (0.37–1.00) *P* = 0.05	0.79 (0.45–1.40) *P* = 0.40	0.66 (0.48–0.89) *P* = 0.008	0.79 (0.57–1.09) *P* = 0.15
Cardiac death	0.70 (0.37–1.34) *P* = 0.29	0.83 (0.40–1.72) *P* = 0.61	NA	NA
SCD	0.59 (0.14–2.44) *P* = 0.46	1.28 (0.26–6.37) *P* = 0.76	NA	NA

TnT pos. population				
Total mortality	0.59 (0.33–1.04) *P* = 0.07	0.82 (0.42–1.61) *P* = 0.56	0.60 (0.41–0.87) *P* = 0.008	0.74 (0.49–1.12) *P* = 0.15
Cardiac death	0.71 (0.36–1.41) *P* = 0.33	1.09 (0.49–2.42) *P* = 0.84	NA	NA
SCD	0.58 (0.14–2.44) *P* = 0.46	1.52 (0.30–7.72) *P* = 0.61	NA	NA

Females				
Total mortality	0.43 (0.19–0.94) *P* = 0.035	0.65 (0.26–1.62) *P* = 0.36	0.51 (0.32–0.83) *P* = 0.006	0.64 (0.38–1.10) *P* = 0.09
Cardiac death	0.35 (0.13–0.98) *P* = 0.046	0.61 (0.19–2.00) *P* = 0.41	NA	NA
SCD	0.0 (0.00-0.00) *P* = 0.96	NA	NA	NA

Males				
Total mortality	0.85 (0.46–1.57) *P* = 0.61	0.81 (0.40–1.65) *P* = 0.56	0.82 (0.56–1.24) *P* = 0.36	0.73 (0.47–1.12) *P* = 0.15
Cardiac death	1.09 (0.46–2.56) *P* = 0.85	0.89 (0.32–2.46) *P* = 0.83	NA	NA
SCD	0.99 (0.20–4.88) *P* = 0.99	1.92 (0.31–12.1) *P* = 0.49	NA	NA

SCD, sudden cardiac death.

^*^Adjusted for gender, age, smoking, hypertension, index diagnosis, diabetes mellitus, CHF (defined by Killip-Kimball class at admission; patients in classes 2 to 4 were classified as CHF patients and those in class 1 as non-CHF), history of previous CHD (i.e., history of either angina pectoris, myocardial infarction, percutaneous coronary intervention, or coronary artery bypass graft), hypercholesterolemia/use of statins, TnT >0.01 ng/mL, eGFR, hsCRP, BNP, body mass index (kg/m^2^), and beta-blockers prior to enrolment.

**Table 5:** The univariate and multivariable HRs (95% CI) for continuous 25(OH)D values.

	2-year follow-up		7-year follow-up	
	Univariate analysis	Multivariable analysis^*^	Univariate analysis	Multivariable analysis^*^
Total population				
Total mortality	0.99 (0.98–1.00) *P* = 0.035	1.00 (0.99–1.01) *P* = 0.38	0.99 (0.99-1.00) *P* = 0.026	1.00 (0.99-1.00) *P* = 0.23
Cardiac death	0.99 (0.98–1.00) *P* = 0.06	0.99 (0.98–1.01) *P* = 0.29	NA	NA
SCD	0.99 (0.97–1.01) *P* = 0.23	1.00 (0.98–1.02) *P* = 0.92	NA	NA

TnT pos. population				
Total mortality	0.99 (0.99-1.00) *P* = 0.012	0.99 (0.98–1.01) *P* = 0.34	0.99 (0.98–1.00) *P* = 0.002	0.99 (0.99-1.00) *P* = 0.07
Cardiac death	0.99 (0.97–1.00) *P* = 0.055	1.00 (0.98–1.01) *P* = 0.54	NA	NA
SCD	0.98 (0.96–1.01) *P* = 0.12	1.0 (0.97–1.02) *P* = 0.74	NA	NA

Females				
Total mortality	0.98 (0.97–1.00) *P* = 0.023	1.00 (0.98–1.01) *P* = 0.53	0.99 (0.98–1.00) *P* = 0.025	1.00 (0.99–1.01) *P* = 0.53
Cardiac death	0.98 (0.97–1.00) *P* = 0.044	0.99 (0.97–1.01) *P* = 0.45	NA	NA
SCD	0.98 (0.95–1.01) *P* = 0.13	0.99 (0.96–1.03) *P* = 0.73	NA	NA

Males				
Total mortality	1.0 (0.98–1.01) *P* = 0.58	0.99 (0.98–1.01) *P* = 0.40	1.0 (0.99–1.01) *P* = 0.42	0.99 (0.99-1.00) *P* = 0.10
Cardiac death	1.0 (0.98–1.01) *P* = 0.65	0.99 (0.97–1.01) *P* = 0.36	NA	NA
SCD	1.0 (0.97–1.03) *P* = 0.99	1.01 (0.98–1.05) *P* = 0.43	NA	NA

SCD, sudden cardiac death.

^*^Adjusted for gender, age, smoking, hypertension, index diagnosis, diabetes mellitus, CHF (defined by Killip-Kimball class at admission; patients in class 2 to 4 were classified as CHF patients and those in class 1 as non CHF), history of previous CHD (i.e., history of either angina pectoris, myocardial infarction, percutaneous coronary intervention, or coronary artery bypass graft), hypercholesterolemia/use of statins, TnT >0.01 ng/mL, eGFR, hsCRP, BNP, body mass index (kg/m^2^), and beta-blockers prior to enrolment.
